# Critical Defect Healing Assessment in Rat Calvaria Filled with Injectable Calcium Phosphate Cement

**DOI:** 10.3390/jfb10020021

**Published:** 2019-05-13

**Authors:** Luis Eduardo Schmidt, Henrique Hadad, Igor Rodrigues de Vasconcelos, Luara Teixeira Colombo, Rodrigo Capalbo da Silva, Ana Flavia Piquera Santos, Lara Cristina Cunha Cervantes, Pier Paolo Poli, Fabrizio Signorino, Carlo Maiorana, Paulo Sérgio Perri de Carvalho, Francisley Ávila Souza

**Affiliations:** 1 Implant Dentistry Post-Graduation Program, São Leopoldo Mandic School of Dentistry and Research Center, Campinas 13.045-755, Brazil; luiz.eduardo.schmidt@gmail.com (L.E.S.); ivasco@hotmail.com (I.R.d.V.); psperri@foa.unesp.br (P.S.P.d.C.); 2 Department of Surgery and Integrated Clinic, Araçatuba Dental of School, São Paulo State University Júlio de Mesquita Filho—UNESP, Araçatuba, São Paulo 16.015.050, Brazil; henriquehadad@gmail.com (H.H.); luara_colombo@hotmail.com (L.T.C.); capalbo.rodrigo@gmail.com (R.C.d.S.); anaflaviaps_06@hotmail.com (A.F.P.S.); laraccerv@gmail.com (L.C.C.C.); 3 Implant Center for Edentulism and Jawbone Atrophies, Maxillofacial Surgery and Odontostomatology Unit, Fondazione IRCSS Cà Granda Maggiore Policlinico Hospital, University of Milan, 47.031 Milan, Italy; pierpaolo.poli@unimi.it (P.P.P.); fabroski@hotmail.it (F.S.); carlo.maiorana@unimi.it (C.M.)

**Keywords:** calcium phosphate cement, biomaterial, bone healing

## Abstract

(1) Background: The tissue engineering field has been working to find biomaterials that mimic the biological properties of autogenous bone grafts. (2) Aim: To evaluate the osteoconduction potential of injectable calcium phosphate cement implanted in critical defects in rat calvaria. (3) Methods: In the calvarial bone of 36 rats, 7-mm diameter critical size defects were performed. Afterwards, the animals were randomly divided into three groups according to filler material: a blood clot group (BC), blood clot membrane group (BCM), and an injectable β-tricalcium phosphate group (HBS) cement group. After periods of 30 and 60 days, the animals were euthanized, the calvaria was isolated, and submitted to a decalcification process for later blades confection. Qualitative and quantitative analysis of the neoformed bone tissue were conducted, and histometric data were statistically analyzed. (4) Results: Sixty days post-surgery, the percentages of neoformed bone were 10.67 ± 5.57 in group BC, 16.71 ± 5.0 in group BCM, and 55.11 ± 13.20 in group HBS. The bone formation values in group HBS were significantly higher (*p* < 0.05) than in groups BC and BCM. (5) Conclusions: Based on these results, it can be concluded that injectable calcium phosphate cement is an osteoconductive material that can be used to fill bone cavities.

## 1. Introduction

Since ancient times, people have been concerned with restoring or replacing damaged parts of bone tissue. Due to trauma and disease, millions of patients worldwide require bone grafting procedures every year. The current types of graft materials have autogenous, homogeneous, and heterogeneous origins, in addition to alloplastic materials [1]. The autogenous bone graft still represents the gold standard because it presents the biological properties of osteoconduction, osteoinduction, and osteogenicity [2]. 

However, autogenous bone grafts have disadvantages like the need for surgical procedures in donor areas, which increase the risks of lesions, infections, hemorrhage, postoperative trauma, morbidity, and poor bone quality and availability [3]. Such disadvantages have been driving researchers to search for a biomaterial that replaces autogenous bone in shape and function [4], preferably synthetic, because it would be free of quantity limitations and contamination risks [5,6].

In recent years, the development of biomaterials used in reconstructive surgeries, especially bone substitutes, has reached a remarkable standard. A biomaterial is considered to be any material of human, animal, plant, or synthetic origin that is intended for implantation in human beings with the prospect of the reconstruction of a specific tissue pattern [7]. Besides, it has to present the following characteristics: it should not induce thrombus formation as a result of the contact between blood and the biomaterial, nor an adverse immune response. Additionally, it has to be non-toxic, non-carcinogenic, and must not disturb the blood flow or produce an acute or chronic inflammatory response that may prevent the differentiation of the adjacent tissues [5]. 

Resulting from all these requirements, a wide range of synthetic materials have been proposed and developed as bone substitutes [8,9]. Among the synthetic materials, calcium phosphate cement was developed in the 1980s [10] and has aroused great interest due to the biocompatibility provided by its excellent biological behavior, bioactivity, and considerable osteoconduction capacity [11]. Calcium phosphate cements (CPCs) exhibit the unique combination of bone conductivity, biocompatibility, and moldability, and they are more interesting than granulated hydroxyapatite ceramics because they can be shaped to fill bone cavities and/or defects [12,13]. 

The term calcium phosphate cement was derived from the fact that this material is prepared by mixing one or more calcium phosphate salts with water or an aqueous solution of calcium phosphate. It may also contain chitosan, alginate, hyaluronate, gelatin, chondroitin sulfate, succinate, or citric acid [14,15,16,17,18,19,20,21]. The mix of the elements forms a cohesive paste which reacts at body or room temperature, resulting in a precipitate with one or more calcium phosphates that hardens through the crossing of these crystals [22]. Calcium phosphate cements are easily manipulated and adaptable materials that can serve as vehicles for the transportation of drugs and medicaments [23]. Their main disadvantage, however, is a low mechanical resistance which can, at best, match the trabecular bone or one fifth of the cortical bone [24,25]. In light of the above, the objective of this study was to evaluate the osteoconduction potential of the injected calcium phosphate cement implanted in critical defects performed in rat calvaria without the interference of epithelial cells and connective tissue through histological and histometric analysis. 

## 2. Material and Methods

This experimental study was performed according to the Ethical Principles for Animal Experimentation adopted by the Brazilian Animal Experimentation Board (COBEA), and was approved by the Animal Ethics and Experimentation Committee (CEEA) of the São Leopoldo Mandic School of Dentistry and Research Center, under Process 2012/0356. The size of the sample was calculated based on a previous publication, with six rats per group (n = 6), in each euthanasia period, totaling 36 animals [26]. 

### 2.1. Animals and Experimental Groups

Thirty-six adult male Wistar rats (Rattus Norvegicus Albinus), weighing between 450 to 500 g were used. The animals were paired at the beginning of the study to reduce the standard deviation. They were then transferred to the facility of the Dentistry School of Araçatuba (FOA, UNESP) where they were kept in individual cages in an air-conditioned environment with standard solid food and water ad libitum throughout the experiment. 

All animals underwent to osteotomy procedure to present a 7-mm wide critical defect and then they were randomly divided into three groups according to the applied filling material: 

*Blood Clot group (BC)*: The surgically created critical defect was filled with a blood clot;

*Blood Clot/membrane group (BCM)*: The critical defect was filled with a blood clot and covered with bovine cortical membrane (Gen Derm, Baumer, São Paulo, Brazil);

*Injectable β-tricalcium phosphate group (HBS) Group*: The critical defect was filled with calcium phosphate cement, based on β-tricalcium phosphate (Graftys BCP^®^, Latin American Solutions (LAS), Brazil) and covered with cortical bovine membrane (Gen Derm, Baumer, São Paulo, Brazil).

### 2.2. Surgical Procedure 

For the surgical step of the experiment, after eight hours of fasting, the animals were submitted to general anesthesia by intramuscular administration of 1% ketamine hydrochloride (Vetaset^®^, Fort Dodge, Saúde Animal LTDA, Campinas, Brazil) at a dosage of 10 mg/kg and 2% xylazine hydrochloride (Dopaser^®^, Laboratory of Brazil Ltd., São Paulo, Brazil) at 5 mg/kg. Post anesthetic induction, a manual trichotomy was performed in the fronto-parietal region with the animals in the ventral decubitus position. Topical povidone-iodine (PVPI) was used for antisepsis of the area (10% PVPI, with 1% active iodine; Riodeine, Rioquímica, São José do Rio Preto, Brazil), followed by the affixing of sterile fields. Surgical access was obtained by a 2 cm sagittal linear incision in the calvarium with a No. 15C scalpel (Feather Industries Ltd., Tokyo, Japan) attached to a No. 3 scalpel handle (Hu-Friedy^®^, Frankfurt, Germany). The flap was detached in its totality, and then lifted and removed with detachers to expose the parietal bone on both sides. 

The osteotomy was performed with a trephine drill, 7 mm internal diameter (Neodent^®^, Curitiba, Paraná, Brazil) coupled to a 16:1 reduction angle reducer (Kavo^®^ do Brasil, Joinvile, Brazil) mounted on a controlled rotation motor (model BLM 600 plus; Driller^®^, Jaguaré, São Paulo, Brazil) at a speed of 1200 rpm, and the osteotomy was performed in the medial region between the parietal lobes, involving the external and internal cortex of the calvarium [27,28]. The osteotomized parietal bone was removed and the dura mater maintained intact. After the critical defect was made, the different filling materials were gently inserted into the critical defect, according to the experimental groups. 

**Figure 1:**
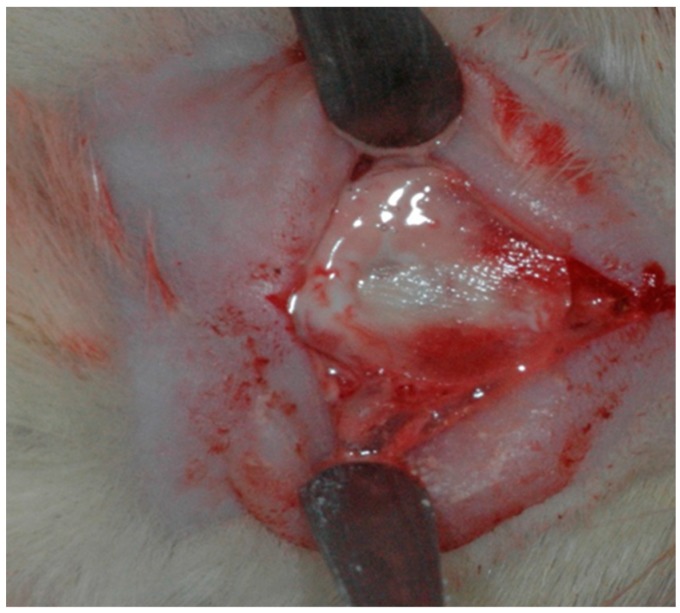
β-tricalcium phosphate cement in the critical size defect.

In group BC, the osteotomy site was irrigated with saline (Darrow, Rio de Janeiro, Brazil), then the filling was inserted delicately into the prepared critical defect. In group BCM, the osteotomy site was also irrigated with saline and then filled with blood and covered with bovine cortical membrane after the blood had clotted (Gen Derm, Baumer, São Paulo, Brazil). In group HBS, the osteotomy site was irrigated with saline, filled with calcium phosphate cement based on β-tricalcium phosphate (Graftys BCP^®^, Latin American Solutions (LAS), Brazil) and covered with cortical bovine membrane (Gen Derm, Baumer, São Paulo, Brazil) (Figure 1). After filling the defects in accordance with the experimental protocol, the whole flap was repositioned and sutured with polyglactin 910 (Vicryl 5-0^®^, Ethicon, Johnson, São José dos Campos, Brazil) using simple interrupted stitches. 

### 2.3. Post-Operative Care 

In the immediate postoperative period, the animals received intramuscular administration of 0.1 mL/kg pentabiotic (Fort Dodge Saúde Animal Ltd., São Paulo, Brazil) and 1 mg/kg/day of sodium dipyrone (Ariston Indústrias Químicas e Farmacêuticas Ltd., São Paulo, Brazil). Both medications were administered at once. 

### 2.4. Euthanasia and Material Collection

At 30- and 60-days post-surgery, the animals were submitted to general intramuscular anesthesia of ketamine hydrochloride and xylazine hydrochloride, as previously described. Next, they were euthanized through perfusion in the left ventricle of 50 mL saline (Darrow, Rio de Janeiro, Brazil) for 10 minutes, followed by 600 mL of 4% formaldehyde (Paraformaldehyde 4% Acros Organics, Geel, Antwerp, Belgium), using a peristaltic perfusion pump (Masterflex^®^ Ls, Cole-Parmer Instrument Company, Vernon Hills, IL, USA) at a rate of 45 mL/min. After euthanasia, the calvaria were carefully removed in block and all of the superficial soft tissue was eliminated. The osteotomies were performed to collect pieces with a margin of 2 cm around the defect. Those pieces were then fixed in 10% neutral buffered formalin (Reagentes Analíticos^®^, Dinâmica Odonto-Hospitalar Ltd., Catanduva, SP, Brazil) and decalcified in 20% EDTA (ethylene diamine tetrameric acid, Merck, Kenilworth, NJ, USA) dissolved in Milli-Q water, with weekly changes for a period of about 14 weeks at room temperature. Subsequently, the material was dehydrated using an increasing and gradual sequence of alcohol (70, 90, 95, and 100) in an orbital shaker (KLine CT-150^®^, Cientec Laboratory Equipment, Piracicaba, SP, Brazil), changing the solution every hour. After these steps, the specimens were diaphanized with xylol and embedded in paraffin to obtain 6 μm wide sections suitable for hematoxylin and eosin (HE) staining (Merck & Co., Inc., Kenilworth, NJ, USA).

### 2.5. Histometric Analysis

Histological analysis was performed by a single investigator, who was blinded with respect to the group allocation. A qualitative histological description was performed for all specimens. The histometric evaluation were made using ImageJ^®^ (U.S. National Institutes of Health, Bethesda, MD, USA), version 3.1, and the area of newly formed bone was expressed in micrometers and converted in percentage as the ratio of newly formed bone area/total area x 100. Five sections from each sample were prepared in a plane parallel to the sagittal suture and through the center of the augmented area and stained with HE. At the surface of the calvaria bone, we determined the newly formed bone inside the calvaria defect without any differences between the groups. Afterwards, measurements were made using an optical microscope (LeicaR^®^ DMLB, Heerbrugg, Switzerland) coupled with a camera Leica DC 300F Microsystems Ltd., Heerbrugg, Switzerland) and connected to a microcomputer Intel core i5 (Dell Computadores do Brasil Ltd., Hortolandia, SP, Brazil). The obtained images were recorded in JPEG format and submitted for analysis. The slides were photomicrographs magnified from the originals by 4× and 40×. The scanned images were recorded in JPEG-format files and analyzed using the “hands free” tools in the ImageJ^®^ (U.S. National Institutes of Health, Bethesda, MD, USA), and then the area of newly formed bone was selected inside all areas of the calvarial defect, even in the area of connective tissue or biomaterial, and blood clot.

### 2.6. Statistical Analysis

The values obtained through histometric analysis were tabulated and submitted to the Shapiro–Wilk test to assess the normality of the distribution. The Mann–Whitney test was used to evaluate bone neoformation in the two experimental periods per group, while the Kruskal–Wallis, followed by Dunn’s post-hoc test, was used to compare the percentages of neoformed bone tissue among the three groups at the same time. All tests were performed using SPSS 17.0 at α = 0.05. 

## 3. Results

### 3.1. Qualitative Histological Analysis

**Figure 2:**
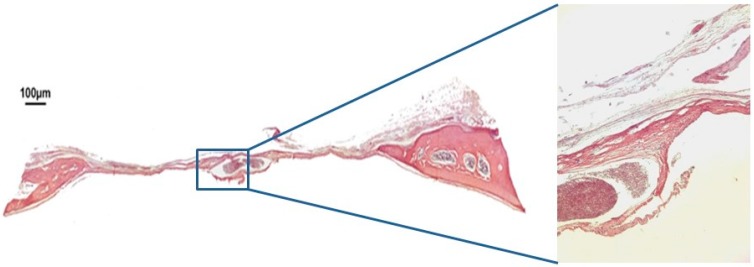
Hematoxylin and eosin staining at 30 days of the clot group BC on 4× and 40×.

**Figure 3:**
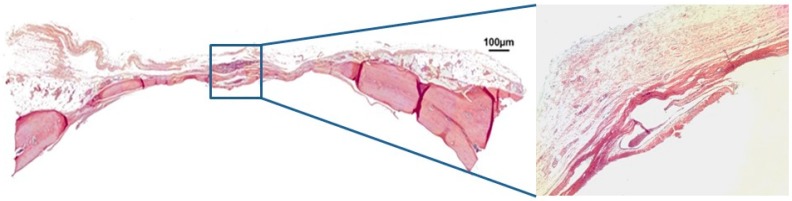
Hematoxylin and eosin staining at 30 days of the clot group (BC) on 4× and 40×.

In group BC, after 30 days, only a small area of neoformed bone was observed, close to the margins of the defect. The space in between was filled with loose, unshaped connective tissue. There was a loss of convex anatomical structure in the cortical walls of the calvaria (Figure 2). After 60 days, the margins of the defect had in fact come closer. However, loose connective tissue and a loss of the calvarium’s convex anatomy were still observed (Figure 3).

**Figure 4:**
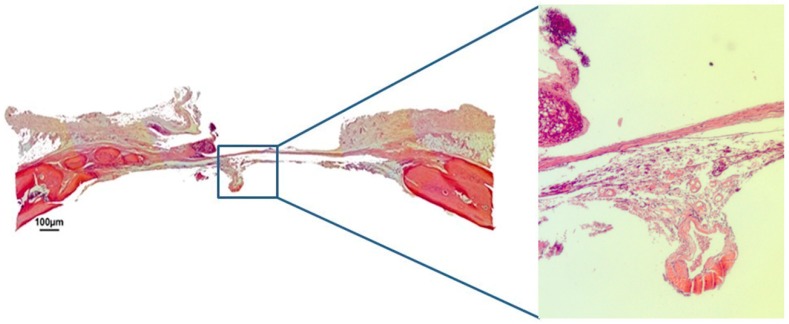
Hematoxylin and eosin staining at 30 days of the clot-membrane group (BCM) on 4× and 40×.

**Figure 5:**
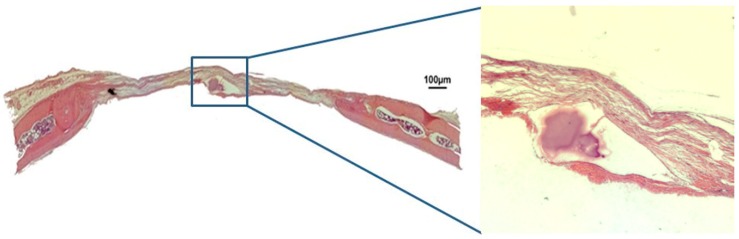
Hematoxylin and eosin staining at 60 days of the clot-membrane group (BCM) on 4× and 40×.

In group BCM at 30 days post-surgery, the bovine cortical membrane was fully reabsorbed, and a large defect filled with loose unshaped connective tissue was still present. The convex anatomical structure of the calvarium’s cortical walls was partially maintained in the defect (Figure 4). After 60 days, fibrous connective tissue was still present between the margins of the defect, while the center of the defect was not closed by new spontaneously formed bone tissue. The convex anatomy of the calvarium’s cortical walls was not maintained (Figure 5).

**Figure 6:**
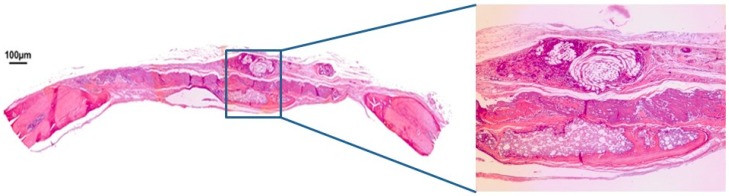
Hematoxylin and eosin staining at 30 days of the injectable β-tricalcium phosphate group (HBS) on 4× and 40×.

**Figure 7:**
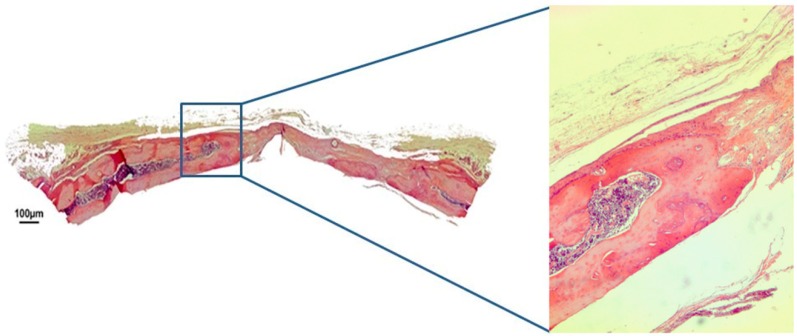
Hematoxylin and eosin staining at 60 days of the injectable β-tricalcium phosphate group (HBS) on 4× and 40×.

In group HBS, after 30 days, the bovine cortical membrane had also been fully reabsorbed, with neoformed bone present in the region of the defect’s margins. In the center of the defect, it was possible to observe some biomaterial particles surrounded by fibroblasts being replaced by mineralized bone tissue, and it was also possible to observe the maintenance of the convex anatomy of the cortical walls of the calvaria (Figure 6). After 60 days post-surgery, the calcium phosphate cement was almost completely reabsorbed, the defect was partially closed by neoformed bone tissue and the convex anatomy of the rat calvaria was maintained (Figure 7).

### 3.2. Histometric Analysis

The mean values of bone formation for the periods of 30 and 60 days were 1.02 ± 0.97 and 10.67 ± 5.57 (Group BC), 6.04 ± 1.69 and 16.71 ± 5.0 (Group BCM), and 9.26 ± 4.82 and 55.11 ± 13.20 (Group HBS), respectively. Intra-group statistical analysis revealed that the mean values of bone formation, in order to close the defect, were significantly higher after 60 postoperative days than at day 30 in all groups. Inter-group analysis, on the other hand, showed that the mean values of bone neoformation in group HBS were substantially higher than those in group BC at 30 and 60 postoperative days, and in group BCM at 60 days post-surgery. The mean values of bone neoformation found in group BCM were significantly higher than those of group BC after 30 days. The mean values, standard deviation, and *p*-values for each group and period are described in Table 1.

The influence of time (in days) on the rate of bone neoformation in each group was also evaluated. It was estimated that the highest rate of increase per day was observed in group HBS at a rate of 1.52 μm^2^ per day (R^2^ = 0.86, *p* < 0.001), followed by group CM at a daily rate of 0.35 μm^2^ per day (R^2^ = 0.73, *p* = 0.001). Finally, group C presented the lowest rate of bone neoformation per day. The rate of bone neoformation in each group and period are described in Table 2. 

## 4. Discussion

The present study proposed to evaluate, through histological and histometric qualitative analysis, the quality of bone repair in critical defects created in rat calvaria using an injectable calcium phosphate cement. It was observed that at 60 postoperative days, the biomaterial was almost completely reabsorbed and the defect closed by neoformed bone tissue for the most part. 

The use of membranes in guided bone regeneration is consolidated in the literature, promoting less substitute bone resorption and preventing the invasion of epithelial cells and connective tissue at the regeneration site [29]. Studies have shown satisfactory results in guided bone regeneration with several types of membrane, with a consensus of its clinical necessity associated with the filling of defects with bone substitutes [30]. The purpose of this study was to evaluate the calcium phosphate cement associated with a commercially available membrane, simulating the clinical situation on critical defects, to evaluate the behavior of the isolated biomaterial without interference from the epithelial cells and connective tissue.

The prospect of having access to an unlimited quantity of bone tissue substitutes [8] without a second surgical area has led researchers to intensify the development of biomaterials that can be used as bone substitutes [31]. These biomaterials added to the patient’s cells and growth factors can improve and accelerate the quality of bone repair [32].

Among all of the synthetic materials used as bone substitutes, calcium phosphate cement has been highly analyzed for its ability to replace human bone, which is a result of its osteoconductive and bioactive proprieties [33], as we observed with the results shown in Table 1. Furthermore, it is considered more attractive than hydroxyapatite granules because it can be modeled to fill cavities, [12,13].

Calcium phosphate cement is prepared by mixing a calcium phosphate salt with water or an aqueous solution. After the dilution of the material, it is possible to obtain a more favorable cellular response, as was verified in a study that evaluated the cytotoxicity of glass ionomer cement (composed of calcium phosphate) in gingival tissue, which showed greater cellular viability, especially of keratinocytes, when the cement was diluted in solutions of approximately 10% [34]. This bioactivity is evidenced due to its main mechanism; ceramics are known for a considerable release of calcium and phosphate ions, which promote the formation of a biological apatite layer, favoring chemotaxis and cell differentiation for a greater bone formation [35] 

Despite its favorable characteristics, its use is largely restricted to situations with smaller loads due to its slow attachment reaction and limited mechanical resistance. The latter can be improved by adding extra components [36] such as macropores [37], which could also contribute to an increased potential for bone neoformation. Calcium phosphate materials exhibit good osseoconduction and osseointegration for bone replacement [38]. A study showed the difference in osteoconductivity and mechanical properties between different sizes of pores of the calcium phosphate cement in bone defects in rabbits, with the group with pores sized between 200–300 µm presenting better properties than other groups with pores of bigger sizes in an earlier period [39], concluding that the porosity of the material influences its osteoconductive and biomechanical potential. In this study, the injectable calcium phosphate cement showed 88% of micro porosity <10 µm, however, even with this particular porosity, the new bone formation was favorable with 55% after the 60 day period. 

These properties were demonstrated by a study evaluating the osteoinduction properties of four different materials, the results showing the ability of calcium phosphate to induce osteoblast differentiation after 14 days of in vitro seeding [40]. In addition, the same study with in vivo analysis demonstrated the ectopic formation of bone tissue in all samples [41]. Even with good results and properties in the procedures for bone grafts, more studies are needed to investigate whether this biomaterial can withstand the mechanical stress of loading [41,42].

When comparing groups BC and BCM, we observed a larger formation in the BCM group, as shown in Table 1. This fact can be explained by the use of a bioabsorbable membrane, which acts as a barrier and prevents unwanted tissues such as connective tissue from occupying the primary tissue space, in the specific case of bone tissue [43]. The use of the blood clot (C) treatment as a control was to prove that there was a critical defect, and that there was no spontaneous repair as the critical size defect cannot heal without the assistance of therapeutic aids or materials designed to encourage bone regeneration [44].

Calcium phosphate cement is a very promising material for bone repair, and in order to improve its performance, it is necessary to develop a ready-made, stable paste with rapid attachment in the defect. In order to increase strength, a so-called “double-setting” cement has been developed, resulting in 150% more resistance when compared to the traditional paste. It contains polypropylene, nylon and carbon fibers, and has a fracture resistance like human cortical bone [42]. The use of additives in calcium phosphate cement, like chitosan, alginate, gelatin, chondroitin sulfate, succinate, or citric acid has also been suggested to improve the material’s qualities, dilute its compounds, and decrease crystal precipitation [14,15,16,17,18,19,20,21]. In this study, the low mechanical strength of the cement did not seem to have interfered in the repair process, considering that in group HBS, the maintenance of the convex architecture of the rat calvaria was observed, as opposed to group C.

Calcium phosphate cement is a versatile biomaterial with a variety of applications and has been employed to carry medication to the site of removed tumors. Therefore, the cement not only functions in facilitating bone formation, but is also a means of keeping medicaments in the region [45,46]. Future studies may uncover ways to further improve the qualities of this biomaterial by assessing its bone neoformation potential when associated with other proven osteoinductive biomaterials such as bone morphogenetic proteins and additional bone growth factors.

## 5. Conclusions

According to the obtained results, it can be concluded that injectable calcium phosphate cement is biocompatible, possesses the biological property of osteoconduction, and can be used for the filling of bone cavities. 

## Figures and Tables

**Table 1:** Mean, standard deviation and comparative analysis of the percentage of bone neoformation in the groups and times evaluated.

Periods	(BC)	(BCM)	(HBS)	*p* Value
	Mean ± SD	Mean ± SD	Mean ± SD	
30 days	1.02 ± 0.97 ^A^	6.04 ± 1.69 ^B^	9.26 ± 4.82 ^B^	0.004 *
60 days	10.67 ± 5.57 ^A^	16.71 ± 5.00 ^AB^	55.11 ± 13.20 ^B^	0.002 *
P value	0.016 *	0.006 *	0.003 *	

SD = standard deviation. Different upper-case letters represent statistical differences between groups at the same time (*p* < 0.05), Kruskal–Wallis test followed by Dunn (between groups at the same time) and Mann–Whitney test (same group at different times). * Statistically significant value.

**Table 2:** Effect of time (in days) through the analysis of linear regression in the percentage of bone neoformation in each group evaluated.

Groups	R^2^	β	*p* Value
Blood Clot Group (BC)	0.57	0.32	0.004 *
Blood Clot Group-Membrane (BCM)	0.73	0.35	0.001 *
Injectable β-TCP group (HBS)	0.86	1.52	<0.001 *

R^2^ = Determination Coefficient. β = Linear Regression Coefficient. * Statistically significant value.
